# Importance of Osmolarity and Oxygen Tension for Cartilage Tissue Engineering

**DOI:** 10.1089/biores.2020.0009

**Published:** 2020-03-31

**Authors:** Stefan Sieber, Martin Michaelis, Hans Gühring, Sven Lindemann, Anne Gigout

**Affiliations:** Osteoarthritis Research, Merck KGaA, Darmstadt, Germany.

**Keywords:** oxygen, osmolarity, cartilage, tissue engineering, chondrocyte

## Abstract

For cartilage repair *in vivo* or evaluation of new therapeutic approaches *in vitro*, the generation of functional cartilage tissue is of crucial importance and can only be achieved if the phenotype of the chondrocytes is preserved. Three-dimensional (3D) cell culture is broadly used for this purpose. However, adapting culture parameters like the oxygen tension or the osmolarity to their physiological values is often omitted. Indeed, articular cartilage is an avascular tissue subjected to reduced oxygen tension and presenting and increased osmolarity compared with most other tissues. In this study, we aimed at evaluating the effect of a physiological oxygen tension (3% instead of 21%) and physiological osmolarity (430 vs. 330 mOsm in nonadjusted DMEM) and the combination of both on the cell proliferation, matrix production, and the phenotype of porcine chondrocytes in a scaffold-free 3D culture system. We observed that a physiological osmolarity had no effect on cell proliferation and matrix production but positively influences the chondrocyte phenotype. A physiological oxygen level prevented cell proliferation but resulted in an increased matrix content/million cells and had a positive influence on the chondrocyte phenotype as well. The strongest benefit was reached with the combination of both physiological osmolarity and oxygen levels; with these conditions, type I collagen expression became undetectable. In addition, at 3% O_2_ the chondrocytes-matrix constructs were found to more closely resemble native cartilage regarding the matrix-to-cell ratio. In conclusion, this study clearly demonstrates the benefit of using physiological oxygen tension and osmolarity in cartilage tissue engineering with the combination of both showing the strongest benefit on the chondrocyte phenotype.

## Introduction

To evaluate new therapeutic compounds *in vitro* and to investigate cellular mechanisms or for tissue engineering, it is of crucial importance to preserve the functional phenotype of the used cells. It can be achieved with culture conditions that are mimicking the physiological environment. For instance, three-dimensional (3D) cell culture—in contrast to monolayer culture—allows the generation of structures resembling their *in vivo* counterparts, regarding cell shape, cellular environment, and cell–cell or cell–matrix interactions.^1^ The benefit of this approach has been demonstrated for many cell types including chondrocytes. In 3D culture, chondrocytes display a similar morphology as in cartilage and produce a cartilage-like extracellular matrix (ECM), rich in type II collagen^2,3^ whereas in monolayer culture they quickly dedifferentiate into fibroblast and a switch from type II to type I collagen expression occurs.^4^ However, even in 3D culture there is some type I collagen produced and the phenotype is not totally preserved especially when serum is being used.^5^

Additional culture parameters can be modulated to imitate the physiological cellular environment *in situ*, like adapting the oxygen tension or the osmolarity. Indeed, articular cartilage is an avascular tissue and oxygen is supplied by diffusion from the synovial fluid resulting in an oxygen tension varying from 9% to 2%.^6^ The chondrocyte metabolism is adapted to low oxygen levels^7^ but typical cell culture is performed at 21% O_2_. Furthermore, the osmolarity in articular cartilage was found to be between 350 and 450 mOsm,^8,9^ whereas typical culture medium as DMEM and HAM'F12 have an osmolarity of ∼330 and 290 mOsm, respectively.

Numerous studies have been performed to evaluate the effect of physiological oxygen tension and osmolarity on chondrocyte phenotype and matrix production. It has been demonstrated that lower oxygen tensions in 3D or monolayer culture (1–5% O_2_) increase the production of cartilage ECM molecule in comparison with cultures at 21% O_2_.^10–12^ In some studies, type I collagen expression was also reduced at low oxygen tensions.^12^ Similarly, more physiological osmolarities (350–400 mOsm) have been shown to increase aggrecan, type II collagen, Sox9 while simultaneously decreasing type I collagen expression.^13^ In addition, at these osmolarities, an increased ^35^S sulfate and ^3^H proline incorporation in monolayer^8^ and glycosaminoglycan (GAG) and ^35^S sulfate incorporation in alginate culture^14,15^ were reported. However, hypertrophy markers were also increased at 410 mOsm in comparison with 310 mOsm in ATDC5 cells^16^ or at 380 mOsm in comparison with 280 mOsm in human OA chondrocytes.^17^ On the contrary, low oxygen levels (2% O_2_) were reported to reduce type X collagen expression in pellet culture of human chondrocytes.^18^

Only little information is available on the combined effect of hypoxia and physiological cartilage osmolarity on chondrocytes. In one study, several culture conditions were tested on bovine chondrocytes in 3D culture including the combination of 5% versus 21% oxygen with a normal or hypertonic culture medium (390 mOsm).^19^ The authors concluded that the combination of a 20% O_2_ tension with a higher osmolarity delivers optimal culture conditions to engineer cartilage tissue.

In this study, we aimed at providing additional insights on the combined effect of oxygen and osmolarity on chondrocytes 3D culture. For this purpose, it was decided to use osmolarity and oxygen conditions at the end of the physiological ranges (3% O_2_ and 430 mOsm) to maximize the probability to observe significant effects. The goals of this study were (1) to evaluate if the benefit of a physiological oxygen tension and a physiological osmolarity are additive on both the phenotype maintenance and ECM deposition; (2) to investigate if a potential increase of hypertrophy markers owing to the higher osmolarity could be compensated by a low oxygen tension; and (3) to compare the matrix-to-cell ratio and the histological appearance of the obtained 3D constructs to those of native cartilage and determine which culture conditions lead to constructs resembling cartilage the most. To do so, porcine chondrocytes were cultured in 3D scaffold-free culture at 330 (nonadjusted DMEM high glucose [DMEMHG] medium) or 430 mOsm and at 3% or 21% O_2_ for 5 weeks. Matrix production, phenotype marker expression, and histology analysis were performed.

## Materials and Methods

### Chondrocyte isolation and culture

Porcine chondrocytes were isolated from the femoral heads of pigs, ∼1 year of age obtained from a local slaughterhouse (Arras, Reichelsheim-Beerfurth). To remove cells from soft tissues, cartilage was digested sequentially with 0.25% w/v collagenase (Cat. No. 17465; Serva GmbH), in HAM's F12 (Cat. No. 21765; Gibco^®^, Life Technologies) for 45 min at room temperature and 0.1% w/v collagenase in HAM's F12 with 1% penicillin/streptomycin (Gibco^®^, Life Technologies) overnight at 37°C. The resulting cell suspension was filtered through 100 μm, then 40 μm cell strainers (Becton Dickinson GmbH), washed several times by centrifugation, and resuspended in culture medium. Isolated porcine chondrocytes were then inoculated at 1 × 10^6^ cells/well in 200 μL of medium in an ultra-low binding 96-well plate to allow the cells to aggregate. Half the samples were incubated at 3% O_2_ and the other half at 21% O_2_. The medium composed of DMEMHG, 10% fetal calf serum (FCS; Promocell GmbH), 50 μg/mL ascorbate-2-phosphate, and 0.4 mM proline with the osmolarity left unchanged (330 mOsm) or adjusted to 430 mOsm with NaCl. After 1 week, the chondrocytes had condensed to form a stable 3D construct and were transferred in a 24-well plate in 1 mL of the same medium or oxygen tension and were cultured for four additional weeks. At the end of the culture period, the 3D constructs were either used for biochemical analysis (DNA, GAG, and hydroxyproline [HPro] content), gene expression (real-time polymerase chain reaction [PCR]), or histological analysis with *n* = 3 for each type of assay. Before biochemical analysis, the constructs were digested overnight at 60°C with papain 0.125 mg/mL (Cat. No. 1.07144.0025; Merck KGaA) in 0.1 M Na_2_HPO_4_, 0.01 M EDTA, and 5 mM l-cysteine.

### Preparation of porcine cartilage samples

For the measurement of GAG, DNA and HPro in native porcine cartilage four femoral heads from four different animals were used. Four pieces of cartilage (∼5 mm in size) were harvested from each femoral head with a scalpeland digested with papain as described previously.

For the histology of native cartilage, 4 mm explants were taken from a femoral heads and cultured 3 days in DMEMHG with 10% FCS (Gibco^®^, Life Technologies), 1% penicillin/streptomycin, 2.5 μg/mL amphotericin B (PAN Biotech GmbH), and 50 μg/mL ascorbate-2-phosphate and one additional day in the same medium with FCS before being processed for Safranin O staining as described in the histology section.

### Biochemical analysis

#### GAG measurement

A dimethylmethylene blue (DMMB) assay was used to quantify GAG in the papain lysate of the 3D constructs. Fifty microliters of the samples (diluted 1/100 in phosphate-buffered saline [PBS]) were mixed with 200 μL of DMMB reagent in a 96-well plate. The absorbance at 525 nm was compared with that of chondroitin sulfate C standards (C4384 diluted in medium or in PBS; Sigma-Aldrich). The DMMB reagent was composed of 16 mg/L DMMB (Applichem) in 0.5% v/v ethanol, 0.2% formic acid, and 2 g/L sodium formate.

#### DNA measurement

DNA was measured with the Quant-iT PicoGreen dsDNA assay kit (Life Technologies). Papain lysates were diluted 1/100 in TE buffer. Twenty microliters of the diluted sample was then mixed with 80 μL TE buffer and 100 μL PicoGreen solution in a black 96-well plate. The fluorescence (Em.:485 nm, Exc.: 535 nm) was measured with a fluorescence reader and compared with that of a DNA standard from 31.25 to 1000 ng/mL diluted from the DNA stock solution provided with the PicoGreen kit. From the DNA concentration the number of cells/construct could be calculated.^20^

#### HPro measurement

The quantitative determination of 4-hydroxyproline was performed using a high-performance liquid chromatography–mass spectrometry/mass spectrometry (HPLC-MS/MS) assay. The stock solution of 4-hydroxyproline (VWR International) was prepared to a concentration of 30 mg/mL in water (Lichrosolv; Merck KGaA) and further diluted to obtain calibration standards ranging from 0.1 to 50.0 μg/mL.

Five microliters of samples was mixed with 10 μL of internal standard (1.2 μg/mL 4-hydroxyproline [^2^H_3_] from C/D/N-Isotopes) and 200 μL hydrochloric acid 25% (v/v) and hydrolyzed overnight at ∼110°C. After centrifugation, samples were evaporated at 55°C/10 Torr (SpeedVac Concentrator Savant SPD131DDA; Thermo Scientific). Samples were then resuspended in 1 mL water and mixed 1/5 in acetonitrile for injection into an HPLC-MS/MS system. HPLC separation (1200 Series HPLC, Agilent; HTC PAL Autosampler, CTC Analytics) was achieved on a hydrophilic interaction chromatography (HILIC) column (Sequant ZIC-HILIC, 50-2.1 mm, 3.5 μm, 200 Å column; Merck KGaA) using a mobile phase gradient (eluent A: 0.1% v/v formic acid in water, eluent B: acetonitrile). Detection was performed on a tandem mass spectrometry (MS, API4000; Sciex) with a turbo ion spray interface operating in positive ion mode. Selectivity of the method was achieved using multiple reaction monitoring for MS/MS detection of the compound and the internal standard. The concentrations of 4-hydroxyproline in unknown samples were calculated by interpolation of the peak area ratio of analyte:internal standard versus the ratio of their nominal concentrations into the regression line obtained from the calibration standards.

### Gene expression

The 3D constructs were placed in a Precellys vial (MK28-R tubes; Bertin Technologies) with 300 μL of RLT buffer (from the RNeasy Mini Kit; Qiagen) and shaken 4 × 1 min at 20 Hz with the Tissue-Lyser (Qiagen). About 590 μL of DEPC-treated water and 10 μL of proteinase K (Qiagen) were then added to the samples and incubated for 10 min at 55°C before being centrifuged 3 min at 10,000 *g*. The supernatant was used for RNA isolation. Cells in monolayer were directly lysed in RLT buffer and directly further processed. RNA isolation was performed with the RNeasy Mini Kit according to the recommendation of the manufacturer. mRNA concentration and quality were analyzed by an Agilent Bioanalyser with an Agilent RNA 6000 Nano Chip (Cat. No. G2938-80023; Agilent Technologies, Inc.).

The reverse transcription was realized with the SuperScript III First-Strand Synthesis SuperMix (Invitrogen Corp.). The cDNA was digested by RNAse H to digest RNA and analyzed by quantitative polymerase chain reaction (qPCR) with the SYBRGreen Jumpstart Taq Ready Mix (Sigma-Aldrich) in the presence of the reverse and forward primer at 200 nM each (ordered from Eurofins MWG Operon; Table 1). Controls contained qPCR master mix without the cDNA template. The reaction was performed in the thermocycler Mx3000P (Agilent Technologies, Inc.). The qPCR reaction was duplicated for each sample.

**Table 1.** List of Primers

Gene	Forward sequence	Reverse sequence
RPL13A	TACGTTCTTTTCCGCCTGCT	TCAAGGTGGTGCGTCTGAAG
Type I collagen	CCTACAGGTACCCTGTGTCC	CCCCAGAAGAACTGGTACAT
Type II collagen	TCTCCAGGTTCTCCTTTCTG	GGATGGGCAGAGGTATAATG
Aggrecan	GATGCTGCTCAGGTGTGACT	GCTTATGCCTTCCCAGCTAC
Sox9	CAGAACTCCGGCTCCTACTA	GGTCTGGTGAGCTGTGTGTA
Runx2	GGGAACTGATAGGGTCCTGA	AGTTCCCAAGCATTTCATCC
Type X collagen	ACCCATTTTCCCCTCTCTTT	AGGAAAACCTGGACAACAGG
Alkaline phosphatase	CCAAAGGCTTCTTCTTGCTG	TGTACCCGCCAAAGGTAAAG

For each sample and each gene, the cycle threshold (Ct) was determined and the relative abundance was calculated according to the following formula: $relative\_abundance=2^{\left(ctHKG-ctGOI\right)}=\frac{2^{ctHKG}}{2^{ctGOI}}$, where HKG is the housekeeping gene ribosomal protein L13A (RLP13A) and GOI, gene of interest. The results are presented as the relative mRNA abundance of the target gene compared with the HKG.

### Histology

Samples were fixed in 4% (w/v) paraformaldehyde, embedded in paraffin. Five micrometers slices were stained with Safranin O 0.25% v/v and Fast Green 0.1% (v/v). The immunohistochemical detection of type I and II collagens (rabbit anti-collagen II antibody, 1:500, ab34712, Abcam and mouse anti-collagen I antibody, 1:200, ab90395, Abcam) was realized using a fully automated immunohistochemistry stainer (Bond III; Leica). To visualize the positive matrix, “mixed red refine” and “mixed DAB refine” were used for type I and type II collagen, respectively. Images were acquired with a Leica SCN400 slide scanner.

### Statistical analysis

Two-way analysis of variance was applied to the data sets using GraphPad Prism software version 7.03 (GraphPad Software, Inc.). Values of *p* ≤ 0.05 were considered statistically significant.

## Results

There are many evidences that removing primary cells from their *in situ* context alters their phenotype and the way they respond to various stimuli. A way to circumvent this is to culture them in an environment mimicking as closely as possible their tissue of origin. In this study, we investigated the impact of physiological oxygen and osmolarity levels on primary chondrocytes in 3D culture.

### Effect of physiological oxygen tension or osmolarity on cell proliferation and ECM deposition

The effect on varying oxygen and osmolarity levels on the cell, GAG, and HPro content in the 3D constructs was investigated. The GAG and HPro contents reflect the proteoglycan and collagen content, respectively.

**FIG. 1.**
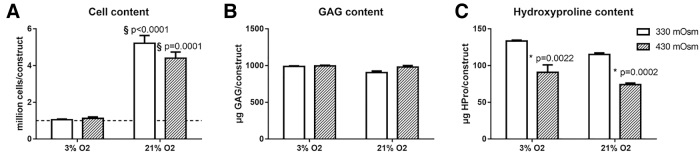
The effect of different oxygen and osmolarity levels on cell proliferation an ECM production. **(A)** Cell content per 3D construct. The dotted line represents the initial number of seeded cells (1 million). **(B)** Amount of μg GAG per 3D construct. **(C)** Amount of μg HPro content per 3D construct. Bars represent the mean of *n* = 3 ± the standard error of the mean. ^§^Difference from 3% oxygen level; *difference from 330 mOsm level. 3D, three dimensional; ECM, extracellular matrix; GAG, glycosaminoglycan; HPro, hydroxyproline.

At an oxygen level of 3%, the cell number in the 3D constructs remained unchanged in comparison with the initial seeding density, whereas a significant cell proliferation was measurable at 21% O_2_ (Fig. 1A). Regarding the GAG content, the amount of GAG per construct was similar in all culture conditions (Fig. 1B) but normalization to the cell number revealed that the GAG content/million cells was significantly higher at lower oxygen levels (Table 2). The HPro content was not altered by the oxygen but was significantly decreased at 430 mOsm in comparison with 330 mOsm (Fig. 1C). Normalization to the cell number showed that the strongest collagen content/million cells occurred at 3% O_2_ and 330 mOsm.

**Table 2.** Amount of Glycosaminoglycan and Collagen in ng/cell for the Four Different Culture Conditions

	3% O_2_, 330 mOsm	3% O_2_, 430 mOsm	21%, 330 mOsm	21%, 430 mOsm	Porcine cartilage^a^
GAG ng/cell	0.94 ± 0.07	0.89 ± 0.1	0.18 ± 0.03	0.22 ± 0.03	1.13 ± 0.34
Collagen^b^ ng/cell	0.38 ± 0.02	0.18 ± 0.04	0.07 ± 0.01	0.04 ± 0.003	2.31 ± 0.9

^a^ Measured from four different animals.

^b^ Calculated with a conversion factor of 7.6.^20^

GAG, glycosaminoglycan.

### The effect of oxygen tension or osmolarity on the chondrocyte phenotype

Because the stability of the chondrocyte phenotype is of utter importance for the generation of a lifelike *in vitro* model, several chondrocyte markers were investigated.

The mRNA levels of the cartilage ECM molecules, type II collagen and aggrecan, the chondrocyte-specific transcription factor Sox9, and the dedifferentiation marker type I collagen were quantified. In addition, the ratio type I collagen/type II collagen expression was calculated.

**FIG. 2.**
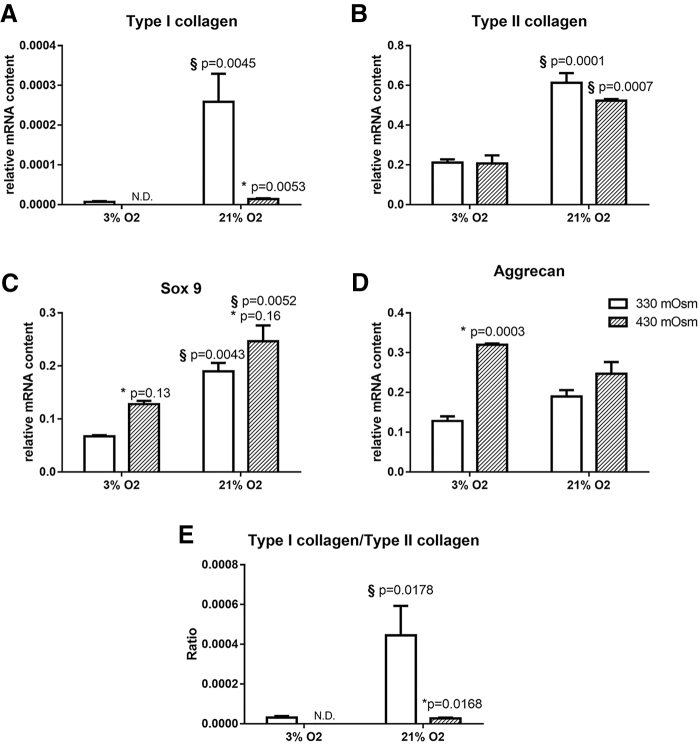
The effect of oxygen and osmolarity on the chondrocyte phenotype. **(A)** Expression of type I collagen, **(B)** type II collagen, **(C)** Sox9, **(D)** aggrecan, and **(E)** the ratio type I collagen/type II collagen expression. N.D. indicates that type I collagen expression was not detectable. Bars represent the mean of *n* = 3 ± the standard error of the mean. ^§^Difference from 3% oxygen level; *difference from 330 mOsm level.

At low oxygen levels, type I and II collagen and Sox9 expressions were significantly reduced (Fig. 2A–C). Especially type I collagen expression could not be detected at 3% O_2_ and 430 mOsm. As a result, at 3% O_2_ the ratio type I collagen/type II collagen was extremely low at 330 mOsm and not detectable at 430 mOsm (Fig. 2E). Regarding aggrecan expression, no significant effect of oxygen could be observed (Fig. 2D).

On the contrary, at high osmolarity, type I collagen expression and the ratio type I collagen/type II collagen expression were significantly decreased at both 3% and 21% O_2_ (Fig. 2A, E). No significant modulation was observable for type II collagen, whereas Sox9 and aggrecan expression were increased at 430 mOsm in comparison with 330 mOsm, both at 3% and 21% O_2_ for Sox9 and at 3% O_2_ only for aggrecan (Fig. 2B–D).

**FIG. 3.**
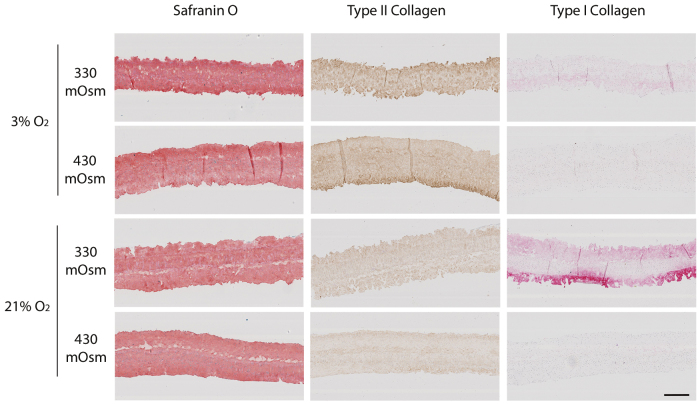
The effect of oxygen and osmolarity on ECM composition. Representative images of Safranin O/Fast Green, type I collagen (mixed red refine) and type II collagen (mixed DAB refine) stainings of constructs cultured at 3% or 21% oxygen and 330 or 430 mOsm. Scale bar is 500 μm.

To evaluate the matrix composition of the 3D constructs, type I and II collagen and Safranin O/Fast Green stainings were performed. Type II collagen and Safranin O/Fast Green stainings were positive in all culture environments. However, the type II collagen staining seems to be weaker at 3% O_2_ compared with 21% O_2_, whereas the contrary was observed at the gene expression level. One possible explanation is that after 5 weeks of culture, when the gene expression analysis was performed, the cells cultured at 3% O_2_ had already accumulated enough type II collagen to initiate a reduction of their type II collagen expression. On the contrary, a positive staining for type I collagen was only observable in 3D constructs cultured at 330 mOsm (Fig. 3). This staining was strong at 21% O_2_ but weak at 3% O_2_. This result is in accordance with the qPCR results (Fig. 2A).

### Effect of oxygen tension or osmolarity on hypertrophy markers

To further evaluate the impact of different oxygen and osmolarity levels, the expression of the hypertrophy markers type X collagen, Runx2 and alkaline phosphatase were investigated. Hypertrophy corresponds to the terminal differentiation of chondrocytes leading to ossification and bone formation during development. However, hypertrophy does normally not occur in mature cartilage, except in OA cartilage, leading to cell apoptosis and matrix calcification that is usually not wanted in a 3D chondrocytes culture model.^21^

**FIG. 4.**
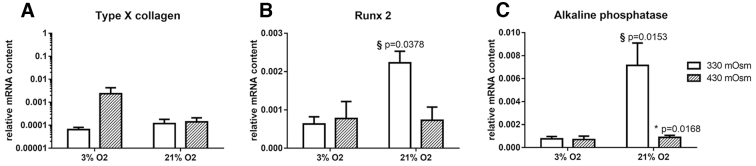
Effect of oxygen and osmolarity on hypertrophy markers. (**A)** Expression of type X collagen, **(B)** Runx2 and **(C)** alkaline phosphatase. Bars represent the mean of *n* = 3 ± the standard error of the mean. ^§^Difference from 3% oxygen level; *difference from 330 mOsm level.

Type X collagen was not influenced by osmolarity or the oxygen tension (Fig. 4A). However, Runx2 and alkaline phosphatase expression were significantly higher at 330 mOsm and 21% O_2_ in comparison with the other culture conditions (Fig. 4B, C). It indicates that both a higher osmolarity or a lower oxygen tension can decrease hypertrophy marker expression.

### Effect of combined high osmolarity and low oxygen on chondrocytes 3D culture

Both a higher osmolarity and a lower oxygen tension were found to have a beneficial effect on the phenotype: Type I collagen expression was decreased and the expression of hypertrophy markers (Figs. 2 and 4). The strongest decrease of type I collagen however was observed at 3% O_2_ together with 430 mOsm. Indeed, with these culture conditions, type I collagen expression was not detectable anymore by qPCR or immunobiological staining (Figs. 2 and 3).

There are several ways to evaluate the quality of engineered cartilage and compare it with native cartilage. For this study, two of the most common approaches were selected: the analysis of the biochemical content and of the histological appearance.^22^ Regarding the matrix-to-cell composition, the cell content was strongly reduced at 3% O_2_ but not the matrix content resulting in higher matrix-to-cell ratios (Table 2). When comparing the composition of the 3D constructs to porcine cartilage, it appears that the matrix-to-cell ratios obtained at 3% O_2_ compared better to with the original tissue than constructs cultured at 21% O_2_. At 3% O_2_, the GAG content/cell was similar at 330 or 430 mOsm but the total collagen content/cell was reduced at 430 mOsm compared with 330 mOsm. It might reflect the fact that type I collagen expression was decreased at 430 mOsm in comparison with 330 mOsm.

**FIG. 5.**
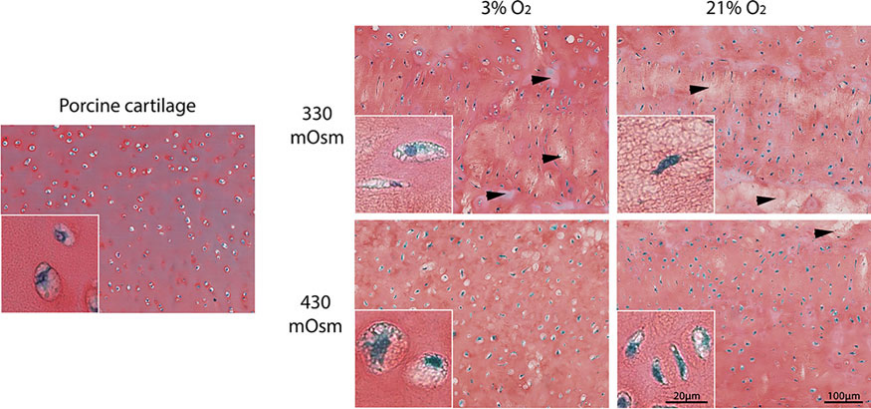
Native porcine cartilage in comparison with cell constructs cultured in various environments. Representative images of Safranin O/Fast Green stainings of native porcine cartilage and constructs cultured at 3% or 21% oxygen and 330 or 430 mOsm. The arrows show areas devoid of Safranin O staining indicating a reduced matrix content. The inserts show the cell morphology in their matrix.

Finally, histological slides of porcine cartilage and the 3D constructs are given in Figure 5. At 330 mOsm it appears that the matrix staining of the 3D constructs is less homogeneous compared with 430 mOsm and with cartilage (white areas in the matrix are marked by arrows on Fig. 5). In addition, cells in the 3D constructs cultured at 3% O_2_ and 430 mOsm seem to present a similar morphology as cells in cartilage; they are round and embedded in a lacuna (see inserts in Fig. 5). This morphology is typical of cartilage cells.^23^ From these qualitative observations, it appears that, the combination of 3% O_2_ together with 430 mOsm resulted in 3D constructs resembling cartilage the most.

## Discussion

In this study we aimed at evaluating if the combination of a physiological osmolarity together with a physiological oxygen tension could be beneficial for chondrocyte 3D culture. For this purpose, porcine chondrocytes were cultured in 3D at 3% or 21% O_2_ and at 330 or 430 mOsm for 5 weeks. The cell and matrix content of the 3D constructs and their histological appearance were evaluated and compared with native cartilage. In addition, the expression of phenotypic markers was investigated.

We observed that applying a physiological oxygen tension or a physiological osmolarity and the combination of both influenced the proliferation, phenotype maintenance, and ECM secretion of the chondrocytes. A higher osmolarity (430 versus 330 mOsm) resulted in a reduced HPro content, reduced type I collagen expression, and an increased Sox9 and aggrecan expression. The reduced HPro content is thought to be mainly driven by the decrease of type I collagen expression as type II collagen expression was not influenced by osmolarity. These results are in accordance with previous findings.^8,13–15^ In addition, it was observed that a higher osmolarity reduces hypertrophy marker expression at 21% O_2_.

Low oxygen resulted in a lower cell content and a decreased type I, type II, and Sox9 expression. However, the overall GAG and HPro content per cell was found to be higher at 3% than at 21% O_2_. There exists contradictory results in literature regarding matrix production in 3D chondrocytes culture at low oxygen versus ambient oxygen tension^24,25^ possibly owing to the different experimental settings. Indeed, the oxygen tension within 3D constructs can be influenced by the oxygen diffusion coefficient in the construct and the oxygen consumption of the cells.^26^ Depending on the type of scaffold used and the cell density, a more or less severe hypoxia can develop within the constructs leading to variable results. However, one reproducible effect was observed in many studies and was observed as well in this study; type I collagen expression was decreased at lower oxygen tensions.^12,18,25^ Finally, similar to Markway et al.,^18^ we observed a lower expression of hypertrophy markers at 3% compared with 21% O_2_.

When comparing the effect of lower oxygen and the effect of a higher osmolarity, both had a positive impact on the chondrocyte phenotype as both strongly downregulated type I collagen expression and the type I collagen/type II collagen ratio. Three percent O_2_ prevented cell proliferation but resulted in an increased matrix production per cell. Regarding the expression of hypertrophy markers, no increase in hypertrophy markers owing to the higher osmolarity, as described previously with ATDC5 or human mesenchymal stem cells,^16^ could be detected. In this study, both a higher osmolarity and hypoxia resulted in lower expression levels of Runx2 and alkaline phosphatase.

To our knowledge, only one study evaluating the combination of physiological oxygen tension and osmolarity on the chondrocytes phenotype and ECM production was published. Ylärinne et al.^19^ cultured bovine chondrocytes in 3D at 5% or 20% O_2_ and unchanged or increased osmolarity (390 mOsm). The authors showed that at 390 mOsm matrix accumulation was increased, whereas 5% O_2_ seemed to be detrimental and concluded that the combination of 20% O_2_ and 390 mOsm provides the optimal conditions for cartilage generation *in vitro*. In contrast to these results, we found that both a lower oxygen tension and higher osmolarity are beneficial for the chondrocyte phenotype with the combination of the two showing the strongest reduction in type I collagen expression (Figs. 2 and 3). In addition, the 3D constructs cultured at low oxygen and high osmolarity appeared to have a cell morphology and a matrix staining resembling native cartilage better than the other 3D constructs (Fig. 5, qualitative assessment). This discrepancy might reside in the fact that we used 1 million cells/construct, whereas they used 6 million cells/construct leading to bigger constructs and possibly anoxia in their center^25^ what can negatively impact matrix production.^27^

The results of this study are highly relevant for the tissue engineering field. To test therapeutic compounds, it is of crucial importance to work with differentiated chondrocytes in a cartilage-like environment. This enables to better predict *in vivo* efficacy and may limit the number of animal studies to be conducted. The field of chondrocyte transplantation for cartilage repair might also benefit from using a lower oxygen and/or a higher osmolarity. Indeed, it is still a challenge to generate hyaline cartilage-like construct for transplantation.^28^ Our culture conditions combining a scaffold-free 3D culture with a more physiological environment that favors the phenotype of the chondrocytes and enables the deposition of a matrix resembling hyaline cartilage can lead to a neotissue with improved functionality; this might ameliorate the ability of the implant to integrate into the surrounding tissue in the cartilage defect and to resist the highly loaded environment of the knee.^22^ Another approach to ameliorate the properties of engineered cartilage constructs is the use of mechanical stimulation or specific growth factors^29^ and it would be of great interest to evaluate their benefit in combination with physiological oxygen and osmolarity levels.

One of the limitations of this study is that the chondrocytes we used originated from young animals and not adult humans. However, because we observed similar effects of osmolarity and oxygen as those reported in human chondrocytes,^13,18^ the combination of both parameters could possibly benefit human chondrocytes 3D culture as well. In addition, to evaluate if the 3D constructs are mechanically functional, it could be of interest to measure the mechanical properties of the 3D cell constructs and compare them with those of native cartilage.^30^

As a conclusion, both physiological osmolarity and oxygen level have a positive effect on the chondrocyte phenotype (decreased expression type I collagen and hypertrophy markers) with the strongest benefit reached with the combination of both (type I collagen expression not detectable anymore). In addition, the use of a physiological oxygen level enables to build chondrocytes-matrix constructs that are the closest to native cartilage regarding the matrix-to-cell ratio.

## Abbreviations Used

3D: three dimensional
DMMB: dimethylmethylene blue
ECM: extracellular matrix
FCS: fetal calf serum
GAG: glycosaminoglycan
GOI: gene of interest
HKG: housekeeping gene
HPLC-MS/MS: high-performance liquid chromatography–mass spectrometry/mass spectrometry
HPro: hydroxyproline
PBS: phosphate-buffered saline
RLP13A: ribosomal protein L13A
